# Family Functioning and Pubertal Maturation in Hispanic/Latino Children from the HCHS/SOL Youth

**DOI:** 10.3390/ijerph22040576

**Published:** 2025-04-07

**Authors:** Ayana K. April-Sanders, Parisa Tehranifar, Mary Beth Terry, Danielle M. Crookes, Carmen R. Isasi, Linda C. Gallo, Lindsay Fernandez-Rhodes, Krista M. Perreira, Martha L. Daviglus, Shakira F. Suglia

**Affiliations:** 1 Department of Biostatistics and Epidemiology, Rutgers School of Public Health, Piscataway, NJ 08854, USA; 2 Department of Epidemiology, Columbia University Mailman School of Public Health, New York, NY 10032, USA; pt140@cumc.columbia.edu (P.T.); mt146@columbia.edu (M.B.T.); 3 Herbert Irving Comprehensive Cancer Center, Columbia University Irving Medical Center, New York, NY 10032, USA; 4 Department of Health Sciences and College of Social Sciences and Humanities, Department of Anthropology and Sociology, Bouvé College of Health Sciences, Northeastern University, Boston, MA 02115, USA; d.crookes@northeastern.edu; 5 Department of Epidemiology and Population Health, Albert Einstein College of Medicine, New York, NY 10461, USA; carmen.isasi@einsteinmed.edu; 6 Department of Pediatrics, Albert Einstein College of Medicine, New York, NY 10461, USA; 7 Department of Psychology, San Diego State University, San Diego, CA 92182, USA; lgallo@sdsu.edu; 8 Department of Biobehavioral Health, College of Health and Human Development, Pennsylvania State University, University Park, PA 16802, USA; fernandez-rhodes@psu.edu; 9 Department of Social Medicine, University of North Carolina School of Medicine, Chapel Hill, NC 27599, USA; krista_perreira@med.unc.edu; 10 Institute of Minority Health Research, University of Illinois College of Medicine, Chicago, IL 60612, USA; daviglus@uic.edu; 11 Department of Epidemiology, Rollins School of Public Health, Emory University, Atlanta, GA 30322, USA; shakira.suglia@emory.edu

**Keywords:** family functioning, childhood adversity, puberty, youth, Hispanic/Latino

## Abstract

Previous studies have examined the association between family dysfunction and pubertal timing in adolescent girls. However, the evidence is lacking on the role of family dysfunction during sensitive developmental periods in both boys and girls from racial and ethnic minority groups. This study aimed to determine the effect of family dysfunction on the timing of pubertal maturation among US Hispanic/Latino children and adolescents. Participants were 1466 youths (50% female; ages 8–16 years) from the Hispanic Community Children’s Health Study/Study of Latino Youth (SOL Youth). Pubertal maturation was measured using self-administered Pubertal Development Scale (PDS) items for boys and girls. Family dysfunction included measures of single-parent family structure, unhealthy family functioning, low parental closeness, and neglectful parenting style. We used multivariable ordinal logistic and linear regression analyses to examine the associations between family dysfunction and pubertal maturation (individual and cumulative measures), with adjustment for childhood BMI and socioeconomic factors, design effects (strata and clustering), and sample weights. Multivariable models of individual PDS items showed that family dysfunction was negatively associated with growth in height (OR = 0.66, 95% CI: 0.44, 0.99) in girls; no associations were found in boys. In the assessment of cumulative PDS scores, family dysfunction was associated with a lower average pubertal maturation score (b = −0.63, 95% CI: −1.21, −0.05) in boys, while no associations were found in girls. Pubertal timing lies at the intersection of associations between childhood adversity and adult health and warrants further investigation to understand the factors affecting timing and differences across sex and sociocultural background.

## 1. Introduction

Different forms of stress in early life/childhood (<18 years) have influenced pubertal timing among youth. Childhood adversities (i.e., maltreatment, abuse, family dysfunction) have typically been associated with accelerating reproductive maturity resulting in early pubertal timing in girls, premature cellular aging, and a general weathering effect of the body across the life course [1] in women. In contrast, little is known about determinants influencing pubertal development in boys. Off-time pubertal maturation, whether early or late, is a public health concern because both have been linked to a range of unfavorable outcomes throughout the life course, including those occurring post-pubertally in later adolescence (e.g., depression, [2] eating disorders, [3] and externalizing psychopathology [4]), and physical outcomes that extend into the midlife and beyond (e.g., obesity, [5] cardiovascular disease [6], and cancer [7]). Therefore, establishing correlates of pubertal maturation could identify relevant modifiable factors associated with later health.

Several developmental theories [8,9,10] have provided a framework for understanding and interpreting the relationship between off-time pubertal maturation explaining the connections between early life stressors, reproductive development, and chronic disease in the midlife. The “evolutionary-developmental theory” [11] is commonly referenced and argues that in response to a stressful or unpredictable family environment, individuals, particularly females, may strategically accelerate their timing of pubertal maturation to increase their survival and reproductive success, beginning sexual activity and reproduction at a relatively early age [9]. However, an adaptive reproductive strategy may induce trade-offs that compromise health and inhibit the reproductive life span. Consistent with the notion of trade-offs due to stress-mediated regulation of life history strategies, early pubertal timing in females is associated with a range of mental and physical health problems, ranging from psychopathology to obesity, CVD, and reproductive cancers [10,12,13].

Alternatively, the “off-time hypothesis” [14] posits that both early and late timing of pubertal maturation produces high levels of distress in a potentially already harsh environment. Evidence from studies of early- and late-maturing youth suggests that being a numerical minority during the adolescent growth period is particularly stressful [15]. In general, pubertal events that occur at expected ages allow adolescents to anticipate, prepare, and learn how to cope with their changing situations [14]. In contrast, developmental events that catch people “off-time” (i.e., either earlier or later than anticipated) make it challenging to adjust [14]. Late-maturing individuals, particularly boys, may experience difficulties maintaining social status among peers and may have less athletic ability than their earlier-maturing peers. Although less frequently studied, evidence suggests that risks accompany later timing of pubertal maturation among boys that include cardiometabolic diseases in adulthood [12,16].

The Adaptive Calibration Model (ACM) [17] provides additional explanatory power by integrating biological mechanisms and evolutionary perspectives into the understanding of pubertal timing. Rather than viewing off-time maturation solely as a social stressor, the ACM suggests that pubertal timing is an adaptive response to early life stressors. The theory posits that the developing stress response system (i.e., hypothalamic–pituitary–adrenal and sympathetic–adreno–medullar) axes) [18] is an underlying mechanism linking pre-pubertal stressful experiences to adolescent and adulthood problems via conditional adaptation of pubertal maturation. Therefore, “calibration” of the stress response system occurs in response to a stressful or unpredictable early life environment and regulates traits and behaviors to optimize the organism’s ability to meet evolutionary goals (e.g., survival and reproductive success). However, while adapting to hostile environments protects the evolutionary function, it may be at the cost of compromised health and inhibited reproductive life span [17]. This mechanistic approach explains why early adversity influences pubertal timing and how it is linked to later health outcomes through stress physiology rather than focusing only on the social adjustment challenges of off-time maturation. Thus, while the Off-Time Hypothesis highlights the immediate social and psychological consequences of pubertal timing, ACM addresses the biological and evolutionary mechanisms that shape these developmental trajectories in response to early life adversity. Building on the framework of the ACM, we use an integrated developmental perspective to guide our understanding of the pathways linking early life stress and pubertal maturation.

Household or family dysfunction is a significant source of early life stress for children that may also impact the timing of pubertal maturation. Evidence from cross-sectional and longitudinal studies has shown that girls who experience early or mid-childhood adversities, such as physical, emotional, and sexual abuse, are more likely to experience earlier pubertal timing and earlier menarche than their peers [19,20,21]. The few studies focused on familial and psychosocial correlates of pubertal timing among boys suggest that stress in the family environment may be associated with off-time pubertal maturation in boys. For instance, a high-stress family environment (i.e., experiencing parental separation or divorce and parent-to-child violence) during childhood was associated with significantly delayed pubertal timing in Turkish boys [20]. In contrast, another study found that Canadian boys whose fathers lacked high school education were more likely to be early maturers [22].

Childhood obesity is also a significant risk factor for off-time pubertal maturation. It is a well-established predictor of earlier pubertal timing in girls [23,24,25], and earlier and later pubertal timing [26,27,28,29] in boys and is influenced by exposure to early life adversities [30]. Evidence shows that higher pre-pubertal body mass index (BMI), a well-established predictor of earlier pubertal timing [24,25], is influenced by exposure to early life adversities [30]. This suggests that BMI may play a mediating role in transmitting adversity-related risk for earlier pubertal timing [31]. However, this potential pathway, an early signal of later cardiometabolic risk and disease, remains poorly understood. A systematic review showed that family functioning is linked with overweight and obesity in children and adolescents [32]. Being overweight or obese in childhood is often correlated with hyperinsulinemia and stimulation of sex hormones and may explain the differential timing of pubertal maturation in males and females. Some studies, however, have found that family functioning or quality of the family environment is associated with pubertal timing independent of childhood body size [19,33,34,35]. These studies focused primarily on age at menarche [19,33,34,35], with limited evidence on other markers of puberty such as breast and pubic hair development in girls [36] and cumulative measures of the maturation of secondary sex characteristics in girls and boys [34]. Whether childhood body size is associated with all milestones of pubertal development and when the associations exist is unknown and warrants further investigation.

The timing of pubertal maturation varies by race and ethnicity, and the role of family functioning is crucial to consider among Hispanic/Latino youth, who face a higher burden of childhood adversities [37] and a higher prevalence of obesity [38,39] than non-Hispanic Whites. US Hispanic/Latino populations are also disproportionately impacted by poverty [40], and lower childhood socioeconomic status has also been linked to earlier puberty [41,42]. Existing research suggests that acculturative stress and intergenerational conflict within Hispanic/Latino families may influence adolescent development, though direct links to pubertal timing remain understudied. Studies indicate that immigrant generation status affects pubertal timing in Latina girls, while acculturation gap conflicts and parental acculturation stress contribute to family dysfunction, which may impact youth development [43,44]. Research on Latino adolescents also highlights how acculturation-related stressors influence behavioral health and family dynamics, which could have indirect effects on pubertal maturation [45]. Further studies are needed to clarify these relationships. Importantly, racial and ethnic minorities in the US, like Black and Hispanic/Latino individuals, have significantly earlier pubertal onset than their White peers [46,47,48]. The interplay of low SES, adverse neighborhood environments, and chronic stressors—including economic hardship and racial discrimination—contributes to disparities in pubertal development among different racial and ethnic groups [49,50]. These factors collectively create a context of chronic stress that can disrupt normal developmental trajectories, underscoring the need for comprehensive strategies to address these intersecting determinants that may drive racial and ethnic disparities in pubertal development.

A few studies [51,52] have tested the effect of childhood adversities on pubertal timing in Hispanic/Latino populations with some mixed findings. Among a cohort of Peruvian girls, exposure to physical and sexual abuse during childhood was associated with early menarcheal age [51]. In a study of Puerto Rican youth, cumulative childhood adversities (a measure that includes household dysfunction) were associated with higher Pubertal Development Scale (PDS) scores (earlier pubertal timing) in girls, but later pubertal timing in boys, compared to unexposed girls and boys, respectively [52]. Finally, early exposure to adverse events (age 0–5 years) was not associated with pubertal onset among a cohort of Mexican–American youth [53]. Still, these findings may not generalize to other Hispanic/Latino heritage groups. The prevalence and experiences of childhood adversities, childhood obesity, and social determinants of health vary across Hispanic/Latino populations and should be examined across the diversity of Hispanic/Latino backgrounds. Additionally, there has not been a specific focus on the family functioning dynamics among Hispanic/Latino households and how they may influence pubertal development among this population. For example, gaps in acculturation between children from immigrant families and their parents may lead to family conflict or poorer family functioning that can adversely affect health behaviors and outcomes [54,55].

This study fills a critical gap in the existing research by examining the relationship between family dysfunction and pubertal maturation among Hispanic/Latino youth, a population that faces a disproportionate burden of childhood adversity, obesity, and socioeconomic disadvantage. While previous research has primarily focused on the effects of early life stress on pubertal timing in girls [19,20,21], this study extends the literature by incorporating boys, addressing the limited empirical evidence on pubertal development in male youth. Additionally, by accounting for childhood BMI, this study provides novel insights into whether obesity mediates the relationship between family dysfunction and pubertal timing, a pathway that remains poorly understood. Importantly, this study leverages data from the Hispanic Community Children’s Health Study/Study of Latino Youth (SOL Youth), allowing for an assessment of these relationships across diverse Hispanic/Latino heritage groups. We hypothesized that family dysfunction in childhood would be significantly associated with earlier pubertal maturation in girls and later pubertal maturation in boys and that childhood overweight/obesity would attenuate the associations observed. This study enhances our understanding of how family functioning, obesity, and socioeconomic factors intersect to influence pubertal development in an underrepresented population, thereby contributing to a more comprehensive framework for identifying modifiable risk factors that may impact long-term health outcomes.

## 2. Materials and Methods

### 2.1. Study Sample

The Hispanic Community Health Study/Study of Latinos (HCSH/SOL) is an ongoing multicenter population-based study designed to examine chronic disease prevalence, incidence, and risk and protective factors in Hispanic/Latino adults (18–74 years at baseline) of diverse backgrounds (Dominican, Puerto Rican, Cuban, Central American, Mexican, South American, Mixed Hispanic, Other) living in four US metropolitan areas (Bronx, NY; Chicago, IL; Miami, FL; and San Diego, CA, USA). The HCHS/SOL sampling design, cohort selection, and study protocol have been reported previously [56,57]. The Hispanic Community Children’s Health Study/Study of Latino Youth (SOL Youth) ancillary study (conducted between 2011 and 2013 and completed in 2014) focused on the offspring (ages 8–16 years) of HCHS/SOL adult participants. Details of recruitment and study design have been previously published [58]. Of the 6741 screened households of HCHS/SOL participants, 1777 eligible children were identified, and 1466 (girls *n* = 738, boys *n* = 728; M = 12 ± 0.1 years [Range: 8–16 years]) were enrolled (2012–2014; (82% participation rate)). Youth and their caregivers completed interview questionnaires assessing social and demographic measures. HCHS/SOL and SOL Youth were approved by the institutional review boards at all participating institutions, and all youth and adults provided informed consent and assent.

### 2.2. Family Dysfunction

Consistent with prior research [59], we combined several youth- and parent/caregiver-reported indicators to create a composite adverse family functioning characteristic score to capture the construct of household and family dysfunction. Where relevant and available, youth-reported measures were chosen to capture the relative perception of the child on the specific family functioning characteristic as opposed to their parent/caregiver, who may have discordant perspectives of their enrolled child. For the composite measure, one point was scored for the presence of each of the following adverse family functioning characteristic items: (1) single-parent family structure (single item, parent/caregiver reported), (2) unhealthy family functioning (youth and parent/caregiver reported), (3) low parental closeness (youth reported), and (4) neglectful parenting (parent/caregiver reported). The summed adverse family functioning characteristic composite score ranged from 0 to 4, with scores of two or more indicating family dysfunction.

The family functioning measures used in SOL Youth were previously validated in English and Spanish. Measures used for this study are described and include a report of Cronbach’s alphas. Scores on the 12-item General Family Functioning (GF12) scale [60] were collected from youth with their parent/caregiver and were used to indicate family functioning (GF12, α_boys_ = 0.74; α_girls_ = 0.79) [61]. The GF12 subscale consists of twelve items, six that reflect healthy family functioning and six that reflect unhealthy functioning [60], and is reliable in clinical and non-clinical settings [61]. Scoring was on a 4-point scale (1 strongly agree to 4 strongly disagree); negatively worded items were reverse-ordered. The scores were summed and then divided by the number of items on the subscale, giving an average score ranging from 1.0 (best functioning) to 4.0 (worst functioning) [62]. Scores < 2 were categorized to indicate “healthy” family functioning and ≥2 to indicate “unhealthy” family functioning [59]. Parental Closeness (concerning mothers and fathers) was assessed using a six-item study-specific measure (PC, α_boys_ = 0.68; α_girls_ = 0.70) completed by youth with the enrolled parent/caregiver. Item responses ranged from 1 (lowest closeness) to 5 (highest closeness) and were averaged and categorized. Informed by approaches in prior research [59], scores above the mean of the summed items (4.48 for boys, 4.36 for girls) were classified as “high” parental closeness, and values at or below the mean as “low” parental closeness. Finally, the enrolled parent/caregiver completed the 16-item Authoritative Parenting Index for each child enrolled in this study. Originally, this measure was developed for reports by children and adolescents [63] but has now been adapted for parent reports [64]. The measure captured parenting styles on two core dimensions: demandingness (setting and enforcing clear limits; monitoring youth behavior and activities) and responsiveness (accepting and affectionate; providing support) (API demandingness, α_boys_ = 0.80; α_girls_ = 0.82; responsiveness, α_boys_ = 0.67; α_girls_ = 0.71). The item responses were summed and averaged with high scores indicating more demandingness or responsiveness (range from 1 to 4). Consistent with prior research [59,63], subscales were dichotomized, using median splits, and combined to create four parenting styles: authoritative (“high” demandingness, “high” responsiveness); authoritarian (“high” demandingness, “low” responsiveness); indulgent (“low” demandingness, “high” responsiveness); and neglectful (“low” demandingness, “low” responsiveness). In analyses, we compared non-neglectful parenting styles (i.e., authoritative, authoritarian, and indulgent; reference category) to the neglectful parenting style and considered the neglectful parenting style as a source of family dysfunction in this capacity. All indicators used to construct the family functioning measure were equally weighted and thus assumed not to carry greater or lesser influence on pubertal development.

### 2.3. Sex

Parents/caregivers reported each child’s sex at recruitment. The response options were “boy” and “girl”. Data on gender identity were not collected.

### 2.4. Pubertal Maturation

Pubertal maturation was assessed using self-administered child-reported scores on the Pubertal Development Scale (PDS) [65]. The PDS is a measure of perceived pubertal development; it has been found to have good reliability and validity and is commonly used in studies of perceived pubertal status. Using longitudinal studies, PDS scores have been shown to reflect the sequence and timing of pubertal events when clinical evaluation is unavailable, such as in our research, and map adequately to those obtained using the Tanner Stages [65,66,67]. The Tanner Staging Scale is a well-established system for identifying stages of sexual maturation (breast, testes, and pubic hair). Staging ranges from no development to full adult maturation. The stagings are assessed via clinician examination and considered the gold-standard method for evaluating pubertal maturation. Agreement between reported self-administered PDS and reported Tanner Scale stages had been observed to be moderate to high, k = 0.50 → 0.70 [68,69], and suggest that self-report indicators of pubertal maturation are acceptable correlates of Tanner stage [66,67,70].

Boys were asked to respond to five questions regarding their pubertal status on growth in height, body hair growth, skin changes, and male-specific development questions about voice deepening and hair growth on the face. For girls, the PDS consisted of six questions about pubertal status: growth in height (i.e., growth spurt), growth of body hair, skin changes, breast growth, menstruation, and age at menarche. Except for the responses on the onset of menarche (yes or no) and age at menarche (age in years), responses to the PDS items were rated on a scale from 1 (“has not begun”) to 4 (“process seems complete”). Modeled cumulatively, PDS scores were normally distributed and ranged from 5 to 20 in boys and girls.

### 2.5. Covariates

Youth age at interview, interview language (English, Spanish), family socioeconomic status [measured by household income < USD 30,000, primary caregiver attained less than high school education or equivalent, and caregiver(s) unemployed], Hispanic/Latino background, nativity (US mainland born, non-US mainland born), field center and BMI percentile were assessed as potential confounders.

### 2.6. Statistical Analyses

All analyses were presented stratified by sex (boys, girls) to account for the distinct natural history of pubertal maturation in males and females, variations in the puberty items ascertained specifically by sex, and the theorized differential effect of family context on pubertal development. Analyses were 2-sided with a type I error set to 5%, accounted for the complex survey design, using appropriate sampling weights, and were performed using SAS SURVEY procedures in SAS 9.4 (SAS Institute, Gary, NC, USA).

We calculated correlation coefficients (Cramer’s V or Pearson’s r) among all demographic, anthropometric, and family functioning characteristics. Variables that were likely to confound the association between family dysfunction and pubertal maturation based on biological plausibility or statistically identified as correlates of measures of family functioning and puberty were included in models; these included youth’s age, nativity, Hispanic/Latino background, study site, BMI percentile, and household socioeconomic factors.

Two series of regression analyses were conducted. Multivariable ordinal logistic regression models were used to examine associations between family dysfunction (1 = yes or 0 = no) and each PDS item. The PDS items had Likert scale responses and the distribution of the responses was non-linear, therefore ordinal logistic regression provided the most efficient and standardized approach for modeling pubertal data [71]. Scores for PDS items were reverse ordered to account for our statistical package modeling probabilities cumulated over the lower ordered values and aid parameter interpretation. Violations of the proportional odds assumption were tested using the score test for the proportional odds assumption [72], and no deviations were observed. In the primary analysis, the outcome variables were PDS scores for growth in height, body hair growth, skin changes, voice change, and facial hair growth in boys. In girls, the outcome variables were PDS score for growth in height, body hair growth, skin changes, and breast development. We also used a multivariable logistic regression model to examine the association between family dysfunction with the onset of menses and linear regression to assess the effect of family dysfunction on the average age at menarche among girls.

In the secondary analysis, we assessed the subset of youth who had complete responses to the PDS variables (486 boys (67% of the total sample) and 480 girls (65% of the full sample)) and summed their PDS scores to create a composite measure for boys and girls. Multivariable linear regression models were used to examine the association between family dysfunction and cumulative PDS scores separately for boys and girls.

For both series of analyses, associations between the individual domains of family dysfunction (i.e., single parent, poor family functioning, low parental closeness, and neglectful parenting) and PDS items were also assessed and data are presented in the Supplement.

## 3. Results

### 3.1. Descriptive Statistics

Participant characteristics by sex are presented in Table 1. The population was 50% girls (*n* = 738), and boys and girls were on average 12.1 ± 0.1 years old at the interview. Most of the youth were interviewed in English (80% boys, 81% girls), were of Mexican background (50% boys, 48% girls), and were born in the US (78% boys and girls). Over two-thirds of the youth had an annual household income of less than USD 30,000, and 38% of boys and 40% of girls had caregivers attaining less than a high school education or equivalent. About 45% of youth had caregivers who were unemployed at the interview. The average BMI percentile in the cohort was 72.5 ± 1.0^,^ and obesity (BMI ≥ 95 percentile) was prevalent, with 29% of boys and 25% of girls.

### 3.2. Family Dysfunction

**Figure 1:**
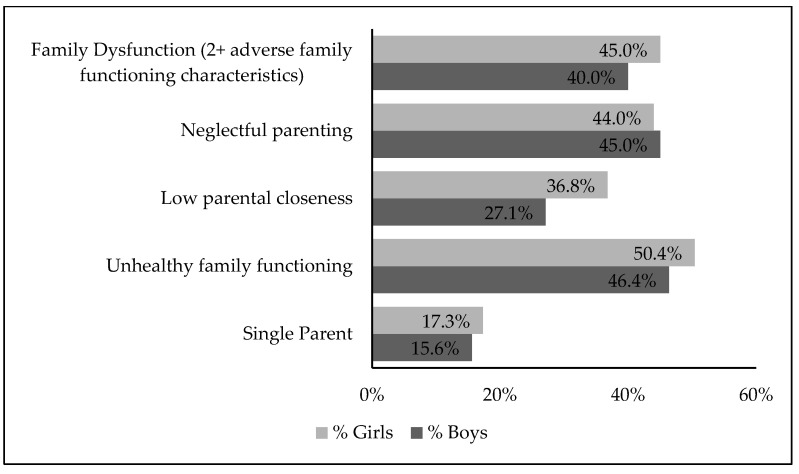
Prevalence of family dysfunction characteristics in all youth, by sex. Reporting source: Single parent—parent-reported; unhealthy family functioning—youth reported; low parental closeness—youth-reported; neglectful parenting—parent-reported.

The distribution of family functioning characteristics is presented in Figure 1. Per caregiver reports, 16% of boys and 17% of girls had single-parent households. Unhealthy family functioning and having a caregiver with a neglectful parenting style were highly prevalent among boys and girls. Girls were more likely to experience one or more family dysfunction characteristics than boys. Forty percent of boys and 45% of girls were categorized as having family dysfunction (two or more adverse family functioning characteristics).

### 3.3. Pubertal Development Scale Scores

Weighted mean PDS scores are reported in Table 2. Boys’ PDS scores ranged from 2.0 ± 0.04 for facial hair growth to 2.7 ± 0.1 for growth in height, suggesting little to some development of the puberty indicators. Girls exhibited more puberty-related development than boys, with weighted mean PDS scores ranging from 2.5 ± 0.04 for skin changes to 3.0 ± 0.1 for growth in height. Among girls with their first menses (53.0%), the mean age at menarche was 11.3 ± 0.1 years.

### 3.4. Multivariable Regression Models—Family Dysfunction and Pubertal Maturation

In the first series of analyses, no significant associations were found between family dysfunction and pubertal maturation estimated by individual PDS score items in boys (Table 3). We found that family dysfunction decreased the odds of growth in height (OR = 0.66, 95% CI: 0.43, 1.03), body hair growth (OR = 0.90, 95% CI: 0.61, 1.34), and voice deepening (OR = 0.79, 95% CI: 0.54, 1.17), and increased the odds of skin changes (OR = 1.06, 95% CI: 0.75, 1.50) and facial hair growth (OR = 1.04, 95% CI: 0.66, 1.64) in fully adjusted models among boys. In girls, family dysfunction was inversely associated with pubertal development for growth in height. In fully adjusted models, the odds of reaching a high score of height maturation (or growth spurt complete) versus combined lower and middle scores decreased by 40% (OR = 0.60, 95% CI: 0.39, 0.90) among those with family dysfunction compared with girls without family dysfunction. Associations between family dysfunction and body hair growth, skin changes, and breast development were in similar directions as the model for height maturation, however, with imprecise confidence intervals. We also did not observe significant associations between family dysfunction and the onset of menses using adjusted binary logistic models (OR = 1.24, 95% CI: 0.57, 2.70), and no significant association between family dysfunction and age at menarche in adjusted linear regression models (b = −0.15, 95% CI: −0.50, 0.20) in girls who reported an onset of menses (Table 3).

In the second series of analyses testing the association between family dysfunction and cumulative PDS score, we found that family dysfunction compared with no family dysfunction was associated with 0.65 points lower pubertal maturation (i.e., later maturation) among boys (Table 4) after adjusting for covariates (b = −0.65, 95%CI: −1.21, −0.10). We found no significant association between family dysfunction and cumulative PDS scores among girls in fully adjusted models (b = −0.34, 95% CI: −0.89, 0.22) (Table 4).

### 3.5. Multivariable Regression Models—Adverse Family Functioning Characteristics (Single Parent, Poor Family Functioning, Low Parental Closeness, Neglectful Parenting) and Pubertal Maturation

The following models tested the effect of individual adverse family functioning characteristics (single-parent household, poor family functioning, low parental closeness, and neglectful parenting) on pubertal maturation in boys (Supplemental Table S1) and girls (Supplemental Table S2A,B) using separate models. Among boys, low parental closeness was marginally associated with skin changes in the age-adjusted model (OR = 1.54, 95% CI: 1.01, 2.36) and fully adjusted model (OR = 1.51, 95% CI: 0.99, 2.30), and neglectful parenting was associated with body hair in the age-adjusted model (OR = 0.46, 0.95), but the association was no longer significant in the fully adjusted model (OR = 0.75, 95% CI: 0.53, 1.07). Among girls, neglectful parenting and low parental closeness suggested delayed height attainment, although with imprecise confidence limits (OR = 0.66, 95% CI: 0.46, 1.04). We observed no significant associations between any of the adverse family functioning characteristics and the onset of menses and age at menarche in girls (Supplemental Table S2B). Finally, in Supplemental Table S3, we present the estimates of cumulative PDS scores for each of the adverse family functioning characteristics. In fully adjusted models, neglectful parenting was significantly associated with delayed pubertal development among boys (b = −0.55, 95% CI: −1.06, −0.04). Other characteristics were not associated with pubertal maturation among boys. None of the characteristics were significantly associated with pubertal maturation among girls.

## 4. Discussion

Using reliable and validated assessments in a targeted population-based study design and accounting for several potential biases, we found some support that family dysfunction contributed to different characteristics of delayed pubertal maturation among Hispanic/Latino girls and boys. These findings add to the limited literature on the etiology of pubertal development and extend existing research on the effects of early life on health across the life course among Hispanic/Latino populations. Specifically, among girls, family dysfunction that accounted for a cumulative effect of multiple adverse family functioning characteristics was associated with delayed height development or growth spurt compared with same-age girls without family dysfunction. Family dysfunction was not linked to individual PDS measures among boys. Still, it was inversely associated with cumulative PDS scores in boys, suggesting that exposure to family dysfunction may delay pubertal maturation compared to same-aged boys without family dysfunction. However, no associations between family dysfunction and cumulative PDS scores were found for girls.

Our study included several domains of family functioning, which are rarely considered together in relation to pubertal timing. Of note, we did not observe an association between family dysfunction and perceived earlier development of breast growth or menarche among female participants. Similar measurements of family functioning were unassociated with pubertal outcomes in a longitudinal study of Mexican–American youth from the Center for the Health Assessment of Mothers and Children of Salinas (CHAMACOS) cohort [53]. It is unclear why there is a lack of effect of family dysfunction with these measures of pubertal development in our study. Previous studies have examined cumulative assessments of adversities, including household or family dysfunction as domains, and have noted earlier and delayed puberty in females [51,52]. Similarly, specific adversity items or dimensions (i.e., physical or sexual abuse, family conflict, mental health disorders, and substance abuse in parents) have been linked to pubertal timing in females [50,73,74,75].

In general, it is challenging to identify specific features of the family environment that are most important to pubertal timing given the wide variation in family contexts hypothesized in this literature. In particular, reliance on secondary data collection to test these relationships results in high variability of measurements, thus limiting standardization and comparability between studies. As this literature continues to actively expand and consider nuances beyond specific, categorized, or aggregated experiences of adversity, dimensions of co-occurrence, such as threat and deprivation, or latent profiles of early life adversity are also being evaluated, particularly with psychopathologic outcomes. These new perspectives must be validated but may offer contextual or environmental information in child development and impact different facets of development beyond what has been observed previously [76,77,78,79]. Further research among Hispanic/Latino youth from diverse socio-cultural and socioeconomic backgrounds may help to elucidate the mechanisms driving associations between family dysfunction and other sources and dimensions of childhood adversity on pubertal development.

Interestingly, we found that girls with family dysfunction were more likely to report their height development as incomplete or in progress than those without. Like infancy and childhood, adolescence represents a period of growth and development that is increasingly vulnerable to stressors. Family dysfunction and other forms of childhood adversities are thought to impact growth directly through modulation of the growth hormone axis or indirectly via other pathways [80,81] and have been linked to slower growth [82] or shorter stature [83] in early childhood. The hormonal mechanism contributing to early maturing girls reporting completeness in height development may be due to the psychological stress response that reduces levels of anabolic hormones like androgens. Androgens promote skeletal growth and lean tissue and are associated with less adipose fat [84]. Exposure to stress like family dysfunction may also be associated with elevated cortisol, which is a direct consequence of stress, and increased insulin production. Reduction in androgens and elevation of cortisol together produce higher levels of fatty tissue [84]. Female puberty is expedited in part due to increased weight gain during adolescence. While earlier age at puberty may result in taller stature during puberty, it can ultimately result in early maturing women attaining shorter adult height than their later maturing peers. In other words, early maturing girls experience a shorter growth period due to a shorter prepubertal growth opportunity and an expedited pubertal development period [85]. At the same time, later maturers have more time for growth during a prolonged prepubertal stage and perceive their height development as incomplete or in progress at later stages of maturity [86]. During puberty, the effects of family dysfunction may cause a period of growth inhibition along with accelerated adipose tissue deposits and visceral adiposity that can accelerate breast development and menarche. The present findings and related studies may indicate important psychosocial and biological mechanisms that need further exploration using repeated height and weight measures to determine the effect of family dysfunction on growth trajectories through adolescence.

There has been relatively limited research on pubertal development in boys. Compared to their female peers who exhibited some to complete development of pubertal indicators, male participants had little to some development, which is generally consistent with youth from other Hispanic/Latino cohorts [52,53] and compared with other racial/ethnic groups [23,31,87]. There was support for our hypothesis on family dysfunction and delayed pubertal maturation for boys. Family dysfunction was associated with delayed pubertal maturation as defined by boys’ overall PDS score. Pubertal maturation among boys has generally been less studied, with only a few recent longitudinal studies assessing this relationship with mixed findings [52,53,88]. Two studies, including Hispanic and non-Hispanic cohorts, have reported that childhood adversities, including household dysfunction, were associated with later pubertal development among boys, which is consistent with our findings [52,88]. However, another study among a Mexican cohort observed no association between family functioning and other measures of childhood adversity and pubertal development in either boys or girls [53]. Evolutionary perspectives point to trade-offs between an individual’s physical development and opportunities to produce offspring [9], which focus on girls with little to no consideration of boys. However, since the cost of reproduction differs by sex, it may be that males and females benefit differentially from environmental exposures and off-time puberty. For instance, early maturation may benefit females but would be associated with smaller physical size, which is counterintuitive to reproductive success which emphasizes power and strength for dominance and status [89]. Another mechanism for future studies is the role of the neuroendocrine system in pubertal development, which may differ by sex, which has been observed in primate studies [90]. One possible explanation is that stress induced by family dysfunction may increase the risk of obesity in childhood and contribute to hypogonadism (failure of the gonads to function correctly, causing a decrease in the production of sex hormones) in prepubertal boys [91].

Our study adjusted for BMI percentile in all analyses but found family dysfunction associated with delayed pubertal development. In early puberty, leptin concentrations increase in both males and females. As one of the hormones directly connected to body fat and obesity, leptin, a hormone released from the fat cells in adipose tissue, sends signals to the hypothalamus in the brain. Typically, early pubertal leptin would decrease in males as puberty progressed, unlike in females [91]. However, with persistent stress, elevated cortisol, and increased adiposity, it is postulated that continually elevated leptin may be responsible for suppressing gonadal function [91]. Alternatively, increased aromatase activity in excessive adipose tissue may cause increased conversion of testosterone to estrogen, increasing the ratio of estrogen to androgens, and may result in suppression of gonadotropins. In both postulates, overweight/obesity is linked to decreased gonadal function and delayed pubertal maturation in boys. As we found that family dysfunction was associated with more delayed pubertal development independent of BMI percentile among boys, it may be that different sources of stress and mechanisms of coping that were not assessed in this study may influence the pathways linking family dysfunction to the timing of puberty in males and should be investigated further among diverse cohorts.

### Strengths and Limitations

This study has several strengths, including a large, multisite cohort representing diverse Hispanic/Latino backgrounds, a detailed characterization of family functioning and social resources, and perceived pubertal development. Several limitations must be considered. Foremost, this study utilized a cross-sectional design, limiting conclusions regarding temporality or drawing causal inferences; further investigations of these associations in longitudinal studies are needed. While we were able to examine a range of family functioning characteristics, the data did not include information regarding other childhood adversities (e.g., maltreatment, violence, bullying) that have been examined previously with pubertal status or information on the duration and severity of the adversity experienced [92].

We assessed pubertal maturation using self-administered PDS scores because clinical examinations were not widely available in the cohort. However, there can be moderate concordance between findings from physical examination and those from the PDS [93], thus allowing for some comparability between this and other studies using physical examinations. Maternal as well as paternal pubertal timing is also known to influence the timing of pubertal development in their offspring [94]. However, this level of data was not available to include in our analyses. Additionally, we modeled pubertal maturation ordinally and linearly, which improves our statistical efficiency and maintains a standardized measure for which associations from this study can be compared to other studies using these measures. Body size may lead to misclassification in self-reporting of pubertal maturity as the excess adiposity can be mistaken for breast development. We also did not have BMI measured before pubertal onset, which limits our inference about the role of adiposity in influencing the timing of pubertal maturation. The role of poverty is also relevant to family functioning, BMI, and pubertal development. While this study accounts for household income based on caregiver reports, multidimensional poverty measures—incorporating various deprivation indicators across health, education, and living standards—may provide a more comprehensive understanding of the impact of poverty on the relationships examined in this study. Finally, the reliability of self-reported PDS may be biased by a child’s desire to over-or underestimate their pubertal maturity compared to their peers. However, in other studies, it has been demonstrated that non-Hispanic white adolescents overestimate their pubertal stage more often than non-white adolescents, who were more likely to underestimate their pubertal stage [95,96].

Despite these limitations, the findings from this study have implications for future research and intervention. Off-time pubertal development, whether earlier or later than peers, produces a high level of stress in a potentially already harsh environment and is a risk factor for physical and mental health issues in adolescence and adulthood [14]. In general, pubertal events that occur at expected ages allow adolescents to anticipate, prepare, and learn how to cope with their changing situations. In contrast, developmental events that catch youth earlier or later than anticipated make it challenging to adjust [14]. Late-maturing individuals, especially boys, may experience difficulties maintaining social status among peers and may have less athletic ability than their earlier-maturing peers. Although less frequently studied, evidence suggests that risks accompanying later timing of pubertal maturation among boys include cardiometabolic diseases, like type 2 diabetes and hypertension, in adulthood [12,16]. Given that family dysfunction was a risk factor for later pubertal timing among youth, clinical providers working with children and adolescents should regularly screen for childhood adversities, mental health symptoms, and elevated cardiometabolic risk factors among youth with off-time pubertal maturation.

The dual role of childhood adversity on pubertal development and later physical and mental health outcomes in males and females from non-white backgrounds is an emerging issue with limited understanding [87]. The average younger age at pubertal maturation among Hispanic/Latino and African American youth compared to their white peers can be an additional burden faced by youth who may carry significant exposure to household dysfunction, abuse, neglect, trauma, racism, and discrimination associated with their SES [24,97]. However, future research is necessary to integrate the separate links between the timing of pubertal maturation, domains of childhood adversity, and adverse health and behaviors. It is important, however, to understand the variability in the effects of childhood adversity on later health outcomes, as not all exposed experience health risks. Sociocultural context (e.g., neighborhood, acculturation, and peer process factors) can be protective among those with off-time puberty. For instance, among girls of Mexican background, White et al. noted that early pubertal timing was associated with depressive symptoms for those living in neighborhoods with lower Hispanic/Latino ethnic density than those in higher density [98]. Similarly, in a study by Mikhail et al., early-maturing girls in disadvantaged neighborhoods exhibited stronger and earlier activation of genetic influences on disordered eating [99]. Further research should consider the interplay between social stress and social context influences pubertal experiences and later health outcomes among youth from racially, ethnically, and socioeconomically diverse communities.

## 5. Conclusions

Studying early life experiences can contribute to understanding factors influencing reproductive development and the potential for increased health risks in later life. Family dysfunction during childhood has a lifelong impact on health for both males and females. This study assessed the timing of pubertal maturation as one such relevant health outcome with consequences for adult health in a large cohort of Hispanic/Latino youth and found that family dysfunction was associated with delayed pubertal maturation, independent of childhood BMI and socioeconomic factors, in both girls and boys.

While findings across a range of studies suggest that variations in girls’ family environment may alter pubertal timing and health implications, the lack of data in boys prevents drawing conclusions about environmental influences on their pubertal timing. This gap in understanding has been a focus of recent work and indicates a need for further research to uncover the relationships and mechanisms linking pubertal development in boys to their mental and physical health across developmental stages. Moreover, there are clear opportunities to delve into the biological nature, mechanisms, and significance of associations observed between early life family environments and pubertal development in humans. Understanding the connections between specific environmental traits and pubertal characteristics, the role of parents/caregivers in creating risk and protective exposures beyond parenting behaviors, and the modifiable factors to mitigate the negative effects of off-time pubertal development as children grow older is relevant and crucial.

These findings highlight the need for interventions that support families at risk of dysfunction to promote healthier developmental trajectories and mitigate long-term health risks associated with off-time pubertal maturation. Family-based programs, such as parenting education and family therapy, can help caregivers foster stable, supportive home environments. Early screening for family dysfunction and atypical pubertal development in pediatric care settings can facilitate timely interventions to address psychosocial stressors. Given this study’s focus on Hispanic/Latino youth, culturally tailored mental health services, bilingual support programs, and community-based resources are essential. Schools can also play a role by integrating health education on puberty, stress management, and coping strategies while offering targeted support through counselors and social workers. The findings underscore the need for longitudinal research to examine how family dysfunction influences pubertal timing and long-term health, particularly in boys, and can inform family-centered policies that enhance economic stability, access to mental health care, and social support. Several longitudinal study designs could effectively track the impact of early-life stressors on child development, particularly pubertal maturation and later health outcomes. These include birth cohorts, cohort-consequential designs, and prospective longitudinal studies with high-frequency assessments. Addressing modifiable factors such as household stress, financial instability, and caregiver relationships can help mitigate the negative effects of early life adversity, ultimately promoting healthier pubertal development and reducing the risk of chronic disease later in life.

## Figures and Tables

**Table 1:** Demographic, family socioeconomic, and body size characteristics of HCHS/SOL Youth children (N = 1466, 2012–2014).

	Boys *n* = 728	Girls *n* = 738
Demographic Characteristics		
Age at Interview	12.1 ± 0.1	12.1 ± 0.1
Language Administered		
English	552 (80.0)	563 (81.0)
Spanish	123 (20.0)	125 (19.0)
Hispanic/Latino background		
Dominican	83 (13.4)	84 (13.4)
Puerto Rican	64 (10.1)	64 (9.7)
Cuban	53 (5.3)	50 (6.0)
Central American	45 (5.0)	67 (7.7)
Mexican	312 (49.6)	336 (47.6)
South American	38 (4.5)	30 (3.8)
Mixed Hispanic	68 (10.1)	67 (10.0)
Other	13 (1.9)	12 (1.8)
Nativity		
Non-US mainland born	165 (22.0)	161 (21.9)
US mainland born	556 (78.0)	572 (78.1)
Family Socioeconomic Characteristics (parent-report)		
Household income less than USD 30,000	517 (72.2)	514 (69.4)
Less than high school or equivalent, caregiver	279 (37.6)	286 (39.7)
Unemployment, caregiver(s)	315 (44.5)	321 (45.6)
Child Body Size		
BMI percentile	72.5 ± 1.5	72.5 ± 1.4
Obesity (BMI percentile ≥ 95th percentile)	230 (28.6)	182 (24.8)

**Table 2:** Pubertal Development Scale (PDS) item scores of HCHS/SOL Youth children (N = 1466).

	Boys	Girls
Puberty Indicators		
Growth in height, (Boys *n* = 563) (Girls *n* = 562)	2.7 ± 0.1	3.0 ± 0.1
1. Has not yet begun to spurt.	89 (13.4)	53 (9.5)
2. Has barely started.	142 (25.5)	130 (20.2)
3. Is definitely underway.	245 (43.0)	204 (35.6)
4. Seems completed.	87 (18.0)	175 (34.7)
Growth in hair, (Boys *n* = 646) (Girls *n* = 651)	2.6 ± 0.04	2.9 ± 0.1
1. Has not yet begun to grow.	116 (16.5)	96 (12.7)
2. Has barely started growing.	154 (22.8)	132 (18.9)
3. Is definitely underway.	263 (40.7)	216 (30.6)
4. Seems completed.	113 (20.0)	207 (37.8)
Skin changes, (Boys *n* = 662) (Girls *n* = 663)	2.3 ± 0.04	2.5 ± 0.04
1. Skin has not yet started changing.	209 (30.0)	161 (23.9)
2. Skin has barely started changing.	173 (23.5)	142 (20.1)
3. Skin changes are definitely underway.	212 (36.9)	265 (38.0)
4. Skin changes seem complete.	68 (9.6)	95 (18.1)
Deepening of voice, (Boys *n* = 672)	2.4 ± 0.1	.
1. Voice has not yet started changing.	209 (27.1)	.
2. Voice has barely started changing.	174 (24.4)	.
3. Voice changes are definitely underway.	192 (32.6)	.
4. Voice changes seem complete.	97 (15.9)	.
Hair growing on face, (Boys *n* = 660)	2.0 ± 0.04	.
1. Facial hair has not started growing.	281 (40.4)	.
2. Facial hair has barely started growing.	195 (30.5)	.
3. Facial hair growth has definitely started.	149 (22.2)	.
4. Facial hair growth seems complete.	35 (6.8)	.
Breast growth, (Girls *n* = 657)	.	2.6 ± 0.04
1. Have not yet started growing.	.	96 (14.3)
2. Have barely started growing.	.	172 (25.6)
3. Breast growth is definitely underway.	.	296 (45.1)
4. Breast growth seems complete.	.	93 (15.0)
Menstruation, (Girls *n* = 722)	.	2.7 ± 0.1
1. No	.	324 (43.1)
2. Yes	.	398 (56.9)
Age at menarche, (Girls *n* = 391)	.	11.3 ± 0.1

Values are presented as mean ± SE or *n* (%) unless otherwise specified. All statistics are weighted.

**Table 3:** Estimated effect (odds ratio (OR) or beta (b), 95% confidence interval (CI)) of maturational development of individual puberty indicators for family dysfunction by sex, SOL Youth, multivariable regression models.

**Models Among Boys**	Growth in Height (*n* = 545)	Body Hair Growth (*n* = 621)	Skin Changes (*n* = 635)	Voice Deepening (*n* = 649)	Facial Hair Growth (*n* = 638)	
	OR (95% CI)	OR (95% CI)	OR (95% CI)	OR (95% CI)	OR (95% CI)	
Family dysfunction						
Model 1	0.68 (0.43, 1.07)	0.90 (0.60, 1.34)	1.12 (0.79, 1.58)	0.77 (0.52, 1.14)	1.03 (0.66, 1.59)	
Model 2	0.68 (0.44, 1.05)	0.91 (0.62, 1.35)	1.08 (0.77, 1.52)	0.79 (0.53, 1.16)	1.05 (0.67, 1.64)	
Model 3	0.68 (0.43, 1.05)	0.92 (0.62, 1.37)	1.07 (0.76, 1.51)	0.79 (0.53, 1.16)	1.06 (0.67, 1.68)	
Model 4	0.66 (0.43, 1.03)	0.90 (0.61, 1.34)	1.06 (0.75, 1.50)	0.79 (0.54, 1.17)	1.04 (0.66, 1.64)	
Models Among Girls	Growth in height (*n* = 549)	Body hair growth (*n* = 632)	Skin changes (*n* = 644)	Breast growth (*n* = 634)	Onset of menses (*n* = 699)	**Age at menarche** **(*n* = 381)**
	OR (95% CI)	OR (95% CI)	OR (95% CI)	OR (95% CI)	OR (95% CI)	b (95% CI)
Family dysfunction						
Model 1	0.69 (0.47, 1.02)	0.83 (0.55, 1.23)	0.84 (0.58, 1.22)	0.85 (0.57, 1.28)	1.22 (0.58, 2.59)	−0.09 (−0.44, 0.27)
Model 2	0.70 (0.47, 1.03)	0.90 (0.60, 1.35)	0.84 (0.57, 1.24)	0.85 (0.56, 1.28)	1.24 (0.53, 2.86)	−0.16 (−0.52, 0.19)
Model 3	0.61 (0.40, 0.93) *	0.91 (0.60, 1.39)	0.81 (0.56, 1.19)	0.88 (0.59, 1.33)	1.28 (0.58, 2.79)	−0.18 (−0.53, 0.17)
Model 4	0.60 (0.39, 0.90) *	0.91 (0.59, 1.38)	0.77 (0.53, 1.13)	0.85 (0.57, 1.27)	1.24 (0.57, 2.70)	−0.15 (−0.50, 0.20)

Model 1: adjusted for youth’s age. Model 2: Model 1 + nativity, Hispanic/Latino background, and field site. Model 3: Model 2 + household socioeconomic factors (incl. parental education, household income, and parental employment status). Model 4: Model 3 + child’s BMI percentile. Abbreviations: OR = odds ratio, CI = confidence interval, SOL = Study of Latinos. Presence of family dysfunction = 2 or more adverse family functioning characteristics. * Denotes two-sided statistical significance at *p* < 0.05.

**Table 4:** Parameter estimates (b, 95% CI) of cumulative pubertal maturation (puberty score) for family dysfunction reported in SOL Youth with complete puberty data, by sex using linear regression models.

Models	Boys (*n* = 473)	Girls (*n* = 472)
	b (95% CI)	b (95% CI)
Puberty score, M (SE)	12.20 (0.22)	14.19 (0.23)
Family dysfunction		
Model 1	−0.60 (−1.21, 0.005)	−0.32 (−0.92, 0.28)
Model 2	−0.63 (−1.21, −0.04) *	−0.31 (−0.91, 0.29)
Model 3	−0.65 (−1.22, −0.07) *	−0.32 (−0.89, 0.25)
Model 4	−0.65 (−1.21, −0.10) *	−0.34 (−0.89, 0.22)

Model 1: adjusted for youth’s age. Model 2: Model 1 + nativity, Hispanic/Latino background, and study site. Model 3: Model 2 + household socioeconomic factors (incl. parental education, household income, and parental employment status). Model 4: Model 3 + child’s BMI percentile. Abbreviation: b = parameter estimate, CI = confidence interval, SOL = Study of Latinos. Presence of family dysfunction = 2 or more adverse family functioning characteristics. * Denotes two-sided statistical significance at *p* < 0.05

## Data Availability

The datasets presented in this article are not readily available because the data are part of an ongoing study. However, anonymized datasets could be accessed upon request for replication studies. Requests to access the datasets should be directed to the Hispanic Community Health Study/Study of Latinos.

## Supplementary Materials

The following supporting information can be downloaded at: https://www.mdpi.com/article/10.3390/ijerph22040576/s1, Supplemental Table S1: Estimated odds ratio (OR, 95% CI) of maturational development of height, body hair growth, skin changes, deepening of voice, and facial hair growth for adverse family functioning characteristics, SOL Youth boys, ordinal logistic regression models; Supplemental Table S2A: Estimated odds ratio (OR, 95% CI) of maturational development of height, body hair growth, skin changes, and breast growth for adverse family functioning characteristics, SOL Youth girls, ordinal logistic regression models; Supplemental Table S2B: Estimated odds ratio (OR, 95% CI) or parameter estimate (b, 95% CI) of onset of menses and age at menarche for adverse family functioning characteristics, SOL Youth girls, logistic and linear regression models; Supplemental Table S3: Parameter estimates (b, 95% CI) of cumulative pubertal maturation (puberty score) for adverse family functioning characteristics reported in SOL Youth with complete puberty data, by sex using linear regression models.
