# Supplementary material for: Family Functioning and Pubertal Maturation in Hispanic/Latino Children from the HCHS/SOL Youth
Source: Int J Environ Res Public Health. 2025 Apr 7;22(4):576. doi: 10.3390/ijerph22040576 (PMC12027471; doi:10.3390/ijerph22040576)
Supplement: Supplementary file 1 [file ijerph-22-00576-s001.zip › ijerph-3411115-supplementary.pdf]

International Journal of
Environmental Research and Public Health

MDPI

Supplemental Table S1. Estimated odds ratio (OR, 95% CI) of maturational development of height, body hair growth, skin changes, deepening of voice, and facial hair growth for adverse family functioning characteristics, SOL Youth boys, ordinal logistic regression models

Models	Growth in height	Body hair growth	Skin changes	Voice deepening	Facial hair growth
	OR (95% CI)	OR (95% CI)	OR (95% CI)	OR (95% CI)	OR (95% CI)
Single Parent	n=545	n=621	n=635	n=649	n=638
Model 1	1.03 (0.56, 1.91)	1.18 (0.67, 2.09)	1.00 (0.52, 1.93)	0.73 (0.45, 1.19)	1.58 (0.90, 2.76)
Model 2	0.95 (0.51, 1.76)	1.01 (0.58, 1.76)	1.06 (0.55, 2.02)	0.69 (0.41, 1.17)	1.47 (0.84, 2.56)
Model 3	0.90 (0.47, 1.69)	1.03 (0.58, 1.83)	1.02 (0.53, 1.99)	0.64 (0.37, 1.11)	1.61 (0.89, 2.90)
Model 4	0.84 (0.45, 1.59)	1.03 (0.58, 1.81)	1.00 (0.51, 1.96)	0.65 (0.38, 1.13)	1.57 (0.88, 2.81)
Poor Family Functioning	n=543	n=617	n=631	n=646	n=634
Model 1	0.98 (0.65, 1.47)	1.02 (0.72, 1.46)	1.29 (0.91, 1.85)	1.05 (0.73, 1.52)	1.02 (0.67, 1.56)
Model 2	0.97 (0.64, 1.45)	0.98 (0.69, 1.40)	1.28 (0.89, 1.83)	1.07 (0.74, 1.53)	1.00 (0.66, 1.52)
Model 3	0.97 (0.65, 1.46)	1.00 (0.70, 1.43)	1.26 (0.87, 1.81)	1.07 (0.75, 1.55)	1.01 (0.66, 1.56)
Model 4	0.89 (0.59, 1.35)	0.97 (0.68, 1.38)	1.21 (0.85, 1.75)	1.08 (0.75, 1.56)	0.98 (0.63, 1.52)
Low Parental Closeness	n=535	n=607	n=623	n=636	n=625
Model 1	1.16 (0.73, 1.83)	1.19 (0.78, 1.84)	1.54 (1.01, 2.36)	1.30 (0.84, 2.00)	1.23 (0.77, 1.96)
Model 2	1.16 (0.73, 1.84)	1.21 (0.79, 1.84)	1.50 (0.98, 2.30)	1.32 (0.85, 2.04)	1.29 (0.81, 2.06)
Model 3	1.14 (0.72, 1.81)	1.22 (0.79, 1.89)	1.49 (0.98, 2.27)	1.31 (0.84, 2.03)	1.32 (0.84, 2.09)
Model 4	1.13 (0.72, 1.77)	1.22 (0.80, 1.88)	1.51 (0.99, 2.30)	1.29 (0.84, 2.00)	1.34 (0.84, 2.15)
Neglectful Parenting	n=545	n=621	n=635	n=649	n=638
Model 1	0.79 (0.53, 1.19)	0.66 (0.46, 0.95)	0.94 (0.65, 1.34)	0.85 (0.58, 1.24)	1.04 (0.72, 1.51)
Model 2	0.84 (0.57, 1.22)	0.75 (0.52, 1.07)	0.91 (0.65, 1.29)	0.85 (0.58, 1.24)	1.10 (0.76, 1.59)
Model 3	0.85 (0.58, 1.24)	0.74 (0.52, 1.06)	0.92 (0.65, 1.30)	0.85 (0.58, 1.25)	1.09 (0.75, 1.57)
Model 4	0.89 (0.62, 1.28)	0.75 (0.53, 1.07)	0.94 (0.67, 1.31)	0.86 (0.58, 1.26)	1.08 (0.75, 1.56)

Model 1: adjusted for youth's age
Model 2: Model 1 + nativity, Hispanic/Latino background, and field site
Model 3: Model 2 + household socioeconomic factors (incl. parental education, household income, and parental employment status)
Model 4: Model 3 + child's BMI percentile
Abbreviations: OR = odds ratio, CI = confidence interval, SOL = Study of Latinos
Presence of family dysfunction = 2 or more adverse family functioning characteristics

*Denotes two-sided statistical significance at P < .05

Supplemental Table S2A. Estimated odds ratio (OR, 95% CI) of maturational development of height, body hair growth, skin changes, and breast growth for adverse family functioning characteristics, SOL Youth girls, ordinal logistic regression models

Models	Growth in height	Body hair growth	Skin changes	Breast growth
	OR (95% CI)	OR (95% CI)	OR (95% CI)	OR (95% CI)
Single Parent	n=548	n=631	n=643	n=633
Model 1	1.29 (0.72, 2.31)	1.06 (0.59, 1.92)	1.20 (0.64, 2.22)	0.95 (0.52, 1.74)
Model 2	1.45 (0.79, 2.66)	0.94 (0.53, 1.70)	1.15 (0.62, 2.14)	0.91 (0.48, 1.72)
Model 3	1.35 (0.71, 2.55)	0.93 (0.50, 1.73)	1.14 (0.62, 2.11)	0.97 (0.50, 1.91)
Model 4	1.38 (0.72, 2.63)	0.95 (0.51, 1.75)	1.17 (0.63, 2.20)	1.03 (0.52, 1.98)
Poor Family Functioning	n=549	n=631	n=644	n=633
Model 1	0.79 (0.54, 1.14)	0.86 (0.59, 1.24)	0.87 (0.60, 1.27)	0.76 (0.50, 1.16)
Model 2	0.83 (0.57, 1.22)	0.88 (0.60, 1.30)	0.86 (0.59, 1.26)	0.75 (0.49, 1.15)
Model 3	0.81 (0.55, 1.19)	0.90 (0.61, 1.33)	0.85 (0.58, 1.24)	0.76 (0.50, 1.15)
Model 4	0.77 (0.52, 1.14)	0.90 (0.61, 1.31)	0.81 (0.55, 1.19)	0.71 (0.46, 1.09)
Low Parental Closeness	n=546	n=629	n=641	n=631
Model 1	0.95 (0.62, 1.46)	1.02 (0.66, 1.57)	0.75 (0.52, 1.09)	1.04 (0.67, 1.62)
Model 2	0.93 (0.61, 1.43)	1.05 (0.68, 1.60)	0.75 (0.51, 1.09)	1.04 (0.67, 1.61)
Model 3	0.87 (0.57, 1.34)	1.06 (0.69, 1.63)	0.73 (0.51, 1.06)	1.09 (0.71, 1.67)
Model 4	0.86 (0.56, 1.33)	1.06 (0.69, 1.62)	0.72 (0.50, 1.03)	1.06 (0.69, 1.62)
Neglectful Parenting	n=547	n=630	n=642	n=632
Model 1	0.78 (0.51, 1.19)	0.81 (0.53, 1.22)	1.08 (0.75, 1.56)	1.12 (0.72, 1.74)
Model 2	0.72 (0.48, 1.08)	0.80 (0.53, 1.23)	1.11 (0.76, 1.62)	1.10 (0.72, 1.70)

Model 3	0.69 (0.45, 1.04)	0.79 (0.51, 1.22)	1.11 (0.76, 1.61)	1.16 (0.76, 1.76)
Model 4	0.69 (0.46, 1.04)	0.78 (0.51, 1.21)	1.06 (0.69, 1.62)	1.13 (0.74, 1.73)

Model 1: adjusted for youth's age
Model 2: Model 1 + nativity, Hispanic/Latino background, and field site
Model 3: Model 2 + household socioeconomic factors (incl. parental education, household income, and parental employment status)
Model 4: Model 3 + child's BMI percentile
Abbreviations: OR = odds ratio, CI = confidence interval, SOL = Study of Latinos
Presence of family dysfunction = 2 or more adverse family functioning characteristics
*Denotes two-sided statistical significance at P < .05

Supplemental Table S2B. Estimated odds ratio (OR, 95% CI) or parameter estimate (b, 95% CI) of onset of menses and age at menarche for adverse family functioning characteristics, SOL Youth girls, logistic and linear regression models*

Models	Onset of menarche	Age at menarche
	OR (95% CI)	b (95% CI)
Single Parent	n=698	n=381
Model 1	0.76 (0.25, 2.29)	-0.23 (-0,67, 0.20)
Model 2	0.60 (0.20, 1.78)	-0.17 (-0.68, 0.33)
Model 3	0.62 (0.20, 1.88)	-0.20 (-0.69, 0.29)
Model 4	0.58 (0.20, 1.69)	-0.25 (-0.69, 0.20)
Poor Family Functioning	n=698	n=381
Model 1	1.11 (0.55, 2.21)	-0.09 (-0.41, 0.23)
Model 2	1.06 (0.50, 2.23)	-0.13 (-0.46, 0.20)
Model 3	1.05 (0.49, 2.24)	-0.11 (-0.44, 0.21)
Model 4	1.01 (0.46, 2.23)	-0.10 (-0.42, 0.22)
Low Parental Closeness	n=696	n=380
Model 1	1.04 (0.50, 2.16)	0.09 (-0.26, 0.45)
Model 2	1.16 (0.55, 2.44)	0.05 (-0.29, 0.40)
Model 3	1.21 (0.60, 2.42)	0.04 (-0.31, 0.39)
Model 4	1.18 (0.61, 2.29)	0.09 (-0.25, 0.43)
Neglectful Parenting	n=697	n=379
Model 1	1.36 (0.71, 2.60)	0.09 (-0.26, 0.44)
Model 2	1.35 (0.67, 2.70)	0.05 (-0.29, 0.39)
Model 3	1.42 (0.75, 2.71)	0.04 (-0.29, 0.36)
Model 4	1.41 (0.75, 2.66)	0.07 (-0.25, 0.39)

Model 1: adjusted for youth's age
Model 2: Model 1 + nativity, Hispanic/Latino background, and field site
Model 3: Model 2 + household socioeconomic factors (incl. parental education, household income, and parental employment status)
Model 4: Model 3 + child’s BMI percentile
Abbreviations: OR = odds ratio, CI = confidence interval, SOL = Study of Latinos
Presence of family dysfunction = 2 or more adverse family functioning characteristics
*Denotes two-sided statistical significance at $P < .05$

Supplemental Table S3. Parameter estimates (b, 95% CI) of cumulative pubertal maturation (puberty score) for adverse family functioning characteristics reported in SOL Youth with complete puberty data, by sex using linear regression models

Models	Boys<br>b (95% CI)	Girls<br>b (95% CI)
Single Parent	n=473	n=471
Model 1	-0.21 (-0.92, 0.49)	-0.14 (-1.32, 1.02)
Model 2	-0.33 (-1.08, 0.42)	-0.12 (-1.21, 0.98)
Model 3	-0.41 (-1.20, 0.39)	-0.13 (-1.31, 1.04)
Model 4	-0.27 (-1.22, 0.34)	0.05 (-1.25, 1.14)
Poor Family Functioning	n=471	n=472
Model 1	0.04 (-0.56, 0.63)	-0.47 (-1.05, 0.12)
Model 2	-0.02 (-0.60, 0.55)	-0.41 (-1.04, 0.21)
Model 3	-0.05 (-0.62, 0.53)	-0.41 (-1.03, 0.21)
Model 4	-0.15 (-0.72, 0.42)	-0.50 (-1.10, 0.11)
Low Parental Closeness	n=465	n=469
Model 1	0.34 (-0.41, 1.08)	-0.05 (-0.66, 0.57)
Model 2	0.36 (-0.41, 1.13)	-0.06 (-0.67, 0.54)
Model 3	0.29 (-0.44, 1.03)	-0.05 (-0.66, 0.56)
Model 4	0.25 (-0.47, 0.97)	-0.08 (-0.63, 0.48)
Neglectful Parenting	n=473	n=470
Model 1	-0.69 (-1.26, -0.13)	0.15 (-0.45, 0.75)
Model 2	-0.64 (-1.19, -0.08)	0.10 (-0.50, 0.70)
Model 3	-0.62 (-1.17, -0.07)	0.09 (-0.48, 0.65)
Model 4	-0.55 (-1.06, -0.04)	-0.02 (-0.53, 0.58)

Model 1: adjusted for youth's age
Model 2: Model 1 + nativity, Hispanic/Latino background, and field site
Model 3: Model 2 + household socioeconomic factors (incl. parental education, household income, and parental employment status)
Model 4: Model 3 + child's BMI percentile
Abbreviations: OR = odds ratio, CI = confidence interval, SOL = Study of Latinos
Presence of family dysfunction = 2 or more adverse family functioning characteristics
*Denotes two-sided statistical significance at $P < .05$
